# Influence of the Hypothalamic Arcuate Nucleus on Intraocular Pressure and the Role of Opioid Peptides

**DOI:** 10.1371/journal.pone.0082315

**Published:** 2014-04-01

**Authors:** Ji Jin, Guo-xu Xu, Zhi-lan Yuan

**Affiliations:** 1 Department of Ophthalmology, the Second Affiliated Hospital of Soochow University, Suzhou, Jiangsu Province, China; 2 Department of Ophthalmology, the First Affiliated Hospital of Nanjing Medical University, Nanjing, Jiangsu Province, China; Univeristy of Melbourne, Australia

## Abstract

**Background:**

An opioid peptide neuron/humoral feedback regulation might be involved in changes of intraocular pressure (IOP). The aims of this study are to investigate the effects of arcuate nucleus (ARC) and opioid peptides on intraocular pressure (IOP).

**Methods:**

Fifty-four healthy purebred New Zealand white rabbits (108eyes) were randomly divided into 4 groups, including control group, electrical stimulation group, [D-Ala2, N-Me-Phe4, Gly5-ol]-enkephalin (DAMGO) group, and [D-Pen 2, D-Pen5]- enkephalin (DPDPE) group. Bilateral IOP was measured after unilateral electrical stimulation of the ARC or unilateral microinjection into the ARC of the selective μ-opioid receptor agonist DAMGO or the selective δ opioid receptor agonist DPDPE, both alone and after pre-administration of either the non-selective opioid receptor antagonist naloxone or saline.

**Results:**

Both electrical stimulation in ARC and micro-injection either <mu> or <delta> opioid receptor agonists, DAMGO or DPDPE, respectively, caused a significant bilateral reduction in IOP (P<0.05) which was more pronounced in the ipsilateral than in the contralateral eye. Pretreatment with naloxone prevented some, but not all IOP reductions.

**Conclusion:**

The ARC takes part in the negative regulation of IOP, an action that may involve opioid neurons.

## Introduction

Although we have not found any published reports describing the following phenomenon, we have observed in our clinical practice that in some patients with open-angle glaucoma, the contralateral intraocular pressure (IOP) increases or decreases after unilateral glaucoma surgery although no obvious changes in other influencing factors have occurred. We therefore considered that surgery causing an IOP decrease in the eye undergoing surgery might cause an imbalance in the previously “balanced” systemic IOP regulation that was then “rebalanced” by adjusting the IOP in the contralateral eye. We also speculated that an opioid peptide neuron/humoral feedback regulation imbalance might be involved in the pathogenesis of glaucoma.

Rabbits are usually chosen for IOP studies because their eyes have the same aqueous outflow channel and the same presence of continuous endothelial cells in the ciliary body as humans, and contain an amount of aqueous humor that is adequate for analysis. Rabbits also have a hypothalamic arcuate nucleus with neurons containing β-endorphin, the major endogenous opioid in the brain, and have opioid peptides present in the eye in the iris and ciliary body [1], [2], [3].

Long-term repeated electrical stimulation of the rabbit hypothalamic ventromedial nucleus increases IOP and the rate of aqueous humor formation, and decreases the aqueous humor outflow rate [4]–[6]. Peripheral electro-acupuncture stimulation of the rabbit sciatic nerve decreases IOP and aqueous humor flow rate and increases aqueous humor endorphin levels, an action prevented by prior iv administration of naloxone [7]. And unilateral topical administration of morphine decreases IOP in both eyes in rabbits [8]. These studies indicate a degree of CNS regulation of IOP, but leave many details unanswered.

The major endogenous opioid peptide in the brain is β-endorphin. The β-endorphin neuron originates in the hypothalamic arcuate nucleus (ARC) [9]. Therefore, it seems possible that opioid neurons originating in the ARC are involved in central bilateral regulation of IOP. The current study explored whether unilateral electrical stimulation of the ARC nucleus would cause bilateral changes in IOP and analyzed possible central and peripheral opioid involvement in such changes.

## Materials and Methods

### Reagents

DAMGO: [D-Ala^2^, N-Me-Phe^4^, Gly^5^-ol]-enkephalin, a μ receptor selective agonist, and DPDPE [D-Pen^2^, D-Pen^5^]-enkephalin, a δ receptor selective agonist, were purchased from Sigma-Aldrich Company, USA. Naloxone, a non-selective opioid receptor antagonist, was purchased from Beijing Sihuan Pharmaceutical Technology Co., Ltd., China. Oxybuprocaine (Benoxil) was purchased from Santen Pharmaceutical Co, Ltd. (Osaka, Japan).

### Experimental animals and grouping

The experiments in this study were approved by the institutional animal use committee of Nanjing Medical University. Healthy purebred New Zealand white rabbits (n = 54, 108 eyes) were provided by Jinling Rabbit Breeding Factory. The rabbits were of either gender, common grade, with body weight ranging from 2.5–3.0 Kg. They were randomly divided into the following 4 groups: Group I, normal control (n = 4); Group II, electrical stimulation with and without interventions (n = 5+4+4+4+4); Group III, DAMGO microinjection into the ARC with and without interventions (n = 4+4+4+3); Group IV, DPDPE microinjection into the ARC with and without interventions (n = 4+4+3+3). Four animals were used for each group (with minor adjustments due to time differences in rabbit purchasing) because this was the number used in a previous report [9]. In all rabbits, both eyes were examined before testing to ensure that the external eye, pupil, and fundus were normal.

### Surgery for ARC electrode implantation

Pentobarbital sodium (3% solution, 1 ml/Kg) was injected into the rabbit's marginal ear vein to induce general anesthesia. Skull drilling and craniotomy were performed at the appropriate locations for stereotaxic instrumentation (left side or right side was randomly selected). Using the stereotaxic apparatus, the ARC was located according to the Paxinos and Watson map, using the following coordinates: Bregma: anterior (A) 0 mm, lateral (L) 1.0–1.15 mm, and height (H) 16.5–17.0 mm. A dual-use electrode for stimulation and injection was inserted and fixed with dental cement. The following electrical stimulation and microinjection experiments were performed on the next day, when the rabbits had regained consciousness.

### Histochemistry

**Figure 1:**
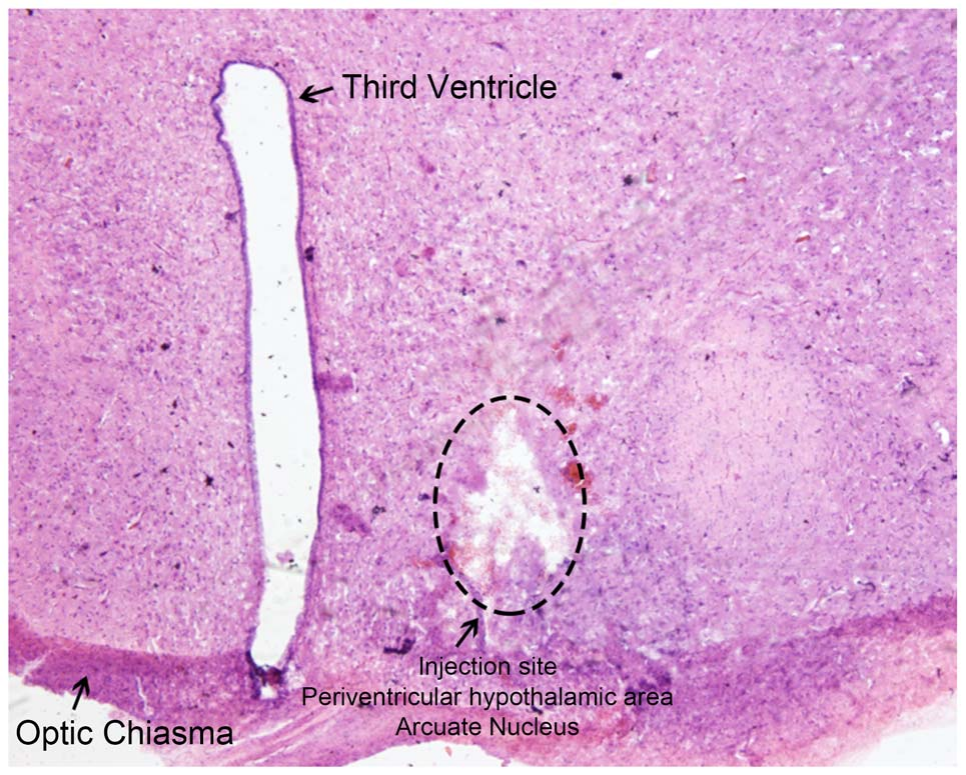
A sample anatomy slice with hematoxylin and eosin stain showing the location of the hypothalamic arcuate nucleus (ARC). A coronal section of hypothalamic regions showing the arcuate nucleus (ARC) (100×).

A portion of the brain underwent histochemical analysis, and 3.5-µm paraffin coronal sections were obtained, dewaxed, and dehydrated. Sections were incubated with hematoxylin and eosin (HE), dehydrated and mounted with neutral gum. Histochemical morphology of hypothalamic areas were observed under 100× magnification with a Leica DM4600 Digital Microscope (Leica Microsystems, Wetzlar, Germany). A sample anatomy slice with HE staining showing the location of the ARC can be seen in Figure 1.

### Electrical stimulation of the ARC

The stimulus electrode was a stainless steel monopolar electrode (0.5 mm diameter) coated with 5–7 layers of insulating paint. The 40 µm tip was bare. A physiological stimulator (KWD-808 Series) was used for the continuous-wave stimulation. The stimulation parameters were 40 µΑ and 70 Hz, and the duration of stimulus was 20 s.

Microinjection in the ARC. The microinjector was introduced into the ARC through the hollow electrode. The volume of drug or saline injected was 0.5 µL, and the injection rate was 0.25 µL/min. The needle was retained in place for 2 min after completion of the injection.

Measurement of IOP. IOP was measured in both eyes in all rabbits before surgery, using the Goldmann tonometer (Topcon Corporation, Tokyo, Japan). This measurement was repeated the day after surgery, when the rabbits were conscious. After electrical stimulation or microinjection of agonist into the ARC, the tonometer was used to measure IOP in both eyes once every 5 minutes for 1 hour and the IOPs recorded. The units were mmHg (1 mmHg = 0.133 kpa). During measurement, one operator fixed the position of the rabbit and a second operator performed the measurement and recorded the data.

### Experimental design

All experiments were performed under the condition of dark adaptation, during a similar time period of the day, and at a similar room temperature. The laboratory was illuminated with red lights [9]. Antagonist concentration was chosen on the basis of work by Bonfiglio et al. [10]

In Group I (4 rabbits), the normal control group, no stimulation was performed. Bilateral IOP was measured and recorded every 5 minutes.

In Group II, the electrical stimulation group, five rabbits underwent electrical stimulation alone; four rabbits underwent electrical stimulation 5 min after 5 µL microinjection of 12.2 mM naloxone into the ARC, and four rabbits underwent electrical stimulation 5 min after microinjection of 5 µL saline into the ARC. In addition, four rabbits underwent a 0.2 ml subconjunctival injection of 12.2 mM naloxone under surface anesthesia, followed by electrical stimulation 5 min later, and four rabbits underwent subconjunctival injection of 0.2 ml saline under surface anesthesia, followed by electrical stimulation 5 min later. Bilateral IOPs were recorded after electrical stimulation once every 5 min in all rabbits.

In Group III, the DAMGO group, three rabbits underwent ARC injection of 2 µmol/µL DAMGO, four rabbits underwent ARC injection of 4 µmol/µL DAMGO, four rabbits underwent bilateral subconjunctival injection of 0.2 ml naloxone (12.2 mM) followed 5 min later by ARC injection of 2 µmol/µL DAMGO, and four rabbits underwent bilateral subconjunctival injection of 0.2 ml saline followed 5 min later by ARC injection of 2 µmol/µL DAMGO. After ARC microinjection, bilateral IOPs were recorded once every 5 min for 1 hour.

In Group IV, the DPDPE group, four rabbits underwent ARC injection of 2 µmol/mL DPDPE, four rabbits underwent ARC injection of 4 µmol/µL DPDPE, three rabbits underwent bilateral subconjunctival injection of 0.2 ml naloxone (12.2 mM) followed 5 min later by ARC injection of 2 µmol/µL DPDPE, and three rabbits underwent bilateral subconjunctival injection of 0.2 ml saline followed by ARC injection of 2 µmol/µL DPDPE. Bilateral IOPs were then recorded once every 5 min for 1 hour.

### Data analysis

Data for intraocular pressure (IOPs) were expressed by mean ± standard error (SE). Due to the repeated measurements of IOP over time, a linear mixed model was applied to investigate the effect of experimental groups (denoted as Group Effect), time after experimental intervention (denoted as Time Effect) and their interaction (denoted as Group×Time) for the ipsilateral eye and contralateral eye, respectively. When main effects or interactions showed significance, further post-hoc multiple comparisons were conducted using a Bonferroni correction to control for overall type I error rates. Analyses are presented as 3 different experimental contexts: (1) electrical stimulation of the ARC, (2) DAMGO injection into the ARC, and (3) DPDPE injection into the ARC. For all 3 contexts, the same animals (n = 4) without any experimental intervention served as the corresponding control group. The statistical analyses were performed with SAS software version 9.2 (SAS Institute Inc., Cary, NC, USA) and the figures were drawn with SPSS statistical software (version 17.0, SPSS Inc., Chicago, IL). A two-tailed *P*<0.05 indicated statistical significance.

## Results

### Number of animal used in each of experiments

Fifty-four animals were used in this study, 4 in the control group, 21 in the electrical stimulation group, 15 in the DAMGO injection group, and 14 in the DPDPE injection group. Details of the group assignments, including the animal number in each subgroup, are shown in Table 1. No rabbit died during or after surgery, and no rabbit had any corneal epithelial lesions that might interfere with the IOP measurements.

**Table 1:** Groups of animals used in each experiment (total n = 54).

Code	Group name	Drug injection	No. of animals
I	I. Control	None	4
II-1	II. Electrical stimulation	None	5
II-2		Injection of naloxone in the ARC (central antagonism)	4
II-3		Injection of saline in the ARC (central blank)	4
II-4		Peripheral subconjunctival injection of 0.2 ml naloxone (peripheral antagonism)	4
II-5		Peripheral subconjunctival injection of 0.2 ml saline (peripheral blank)	4
III-1	III. DAMGO injection	Injection of DAMGO in the ARC (2 µmol/µl)	4
III-2		Injection of DAMGO in the ARC (4 µmol/µl)	4
III-3		Subconjunctival injection of 0.2 ml naloxone+injection of DAMGO in the ARC (2 µmol/µl)	4
		(peripheral antagonism)	
III-4		Subconjunctival injection of 0.2 ml saline+injection of DAMGO in the ARC (2 µmol/µl) (peripheral blank)	3
IV-1	IV. DPDPE injection	Injection of DPDPE in the ARC (2 µmol/µl)	4
IV-2		Injection of DPDPE in the ARC (4 µmol/µl)	4
IV-3		Subconjunctival injection of 0.2 ml naloxone+injection of DPDPE in the ARC (2 µmol/µl)	3
		(peripheral antagonism)	
IV-4		Subconjunctival injection of 0.2 ml saline+injection of DPDPE in the ARC (2 µmol/µl) (peripheral blank)	3

### Effect on IOP of unilateral electrical stimulation of the ARC

**Figure 2:**
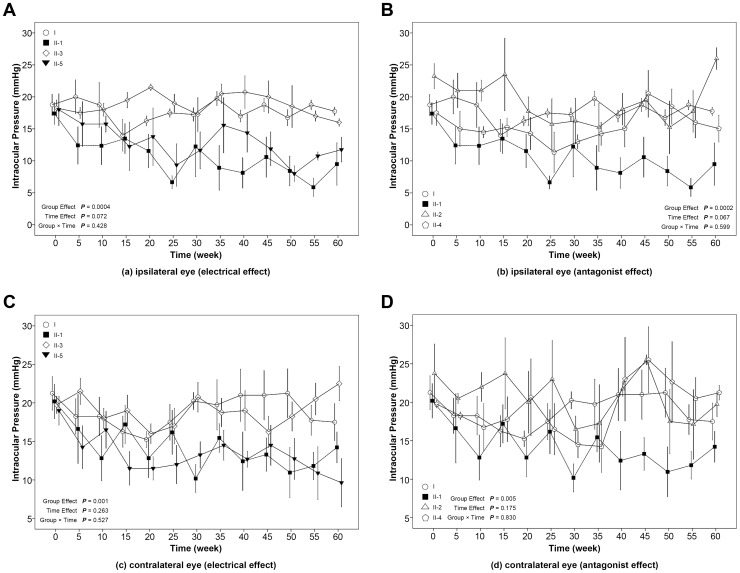
The effect of unilateral electrical stimulation of the ARC on intraocular pressure for the ipsilateral eye on electrical (a) and antagonist (b) effects, and for the contralateral eye on electrical (c) and in antagonist (d) effects. The error-bar (mean ± SE) of each group was located at time points of 0, 5, 10, 15, 20, 25, 30, 35, 40, 45, 50, 55 and 60 minutes, respectively. A slight separation was made for error-bars at the same time points to avoid overlapping. I, Control group; II-1, electrical stimulation group; II-2, ARC naloxone+electrical stimulation group; II-3, ARC saline+electrical stimulation group; II-4, subconjunctival naloxone+electrical stimulation group; II-5, subconjunctival saline+electrical stimulation group.

In the ipsilateral eye after unilateral stimulation of the ARC, significant group effects were seen on IOP for both the electrical (Figure 2a, *P* = 0.0004) and antagonist (Figure 2b, *P* = 0.0002) effects. After Bonferroni correction, the overall group means showed statistically significant differences between the electrical stimulation group and the control group (mean difference = −7.21 mmHg; *P* = 0.0016), the ARC naloxone injection group (mean difference = −8.68 mmHg; *P* = 0.0002), and the ARC saline injection group (mean difference = −8.17 mmHg; *P* = 0.0004). Significant differences in the group means were also seen between the subconjunctival saline group and the ARC naloxone (mean difference = −6.27 mmHg; *P* = 0.011) and ARC saline (mean difference = −5.77 mmHg; *P* = 0.023) groups. When data from the 35–60 min time period were averaged and compared (that is, when responses have reached a quasi-steady state and less variability between measurements at successive time points is seen), subconjunctival naloxone was seen to prevent the stimulation-induced decreased in IOP (Table 2). In other words, electrical stimulation of the ARC significantly decreased IOP (mean difference = 9.56 mmHg; *P* = 0.009), and this decrease was lessened by centrally injected naloxone and by locally injected naloxone (at times later than 30 min), and by saline when injected into the ARC, but not when injected subconjunctivally.

**Table 2:** Intraocular Pressure during the 35 to 60 min time period.

	Control	Stim	2 DAMGO	4 DAMGO	2 DPDPE	4 DPDPE
***Ipsilateral Eye***						
No Nal	18.13 (1.74)^a^	8.57 (1.55)*	7.55 (0.85)*	3.63 (0.85)*	8.52 (0.88)*	5.34 (0.88)*
ARC Nal		18.64 (1.74)				
ARC Sal		18.79 (1.74)				
Subc Nal		16.56 (1.74)	9.99 (0.85)*		12.72 (1.02)*	
Subc Sal		12.05 (1.74)	3.78 (0.98)*		6.49 (1.02)*	
***Contralateral Eye***						
No Nal	19.71 (1.83)	13.03 (1.64)	11.27 (1.59)*	7.23 (1.59)*	6.55 (1.66)*	12.21 (1.66)
ARC Nal		19.81 (1.83)				
ARC Sal		19.21 (1.83)				
Subc Nal		21.19 (1.83)	13.39 (1.59)		14.56 (1.92)	
Subc Sal		12.50 (1.83)	7.86 (1.83)*		9.17 (1.92)*	

a Least-squares mean (LS-mean) with corresponding standard error (SE) using the linear mixed model was presented.

* P<0.05 compared to Group I (Control).

In the contralateral eye after unilateral stimulation of the ARC, significant group effects were also seen for both electrical (Figure 2c, P = 0.001) and antagonist (Figure 2d, P = 0.005) effects. Statistically significant differences in the overall group means were seen between the following groups: control group and subconjunctival saline group (mean difference = −5.56 mmHg; *P* = 0.026); electrical stimulation group and both the ARC (mean difference = −6.48 mmHg; *P* = 0.004) and the subconjunctival (mean difference = −5.17 mmHg; *P* = 0.031) naloxone groups; ARC naloxone group and subconjunctival saline group (mean difference = −7.34 mmHg; *P* = 0.002); ARC saline group and subconjunctival saline group (mean difference = 5.71 mmHg; *P* = 0.021); subconjunctival naloxone group and subconjunctival saline group (mean difference = 6.03 mmHg; *P* = 0.0131). Using data from the 35–60 min time period (Table 2), one can see that in the contralateral eye, electrical stimulation also lowers IOP although not to a statistically significant degree (compared to control, mean difference: −6.68, *P* = 0.203), and ARC naloxone, subconjunctival naloxone, and ARC saline increase IOP. In other words, although the overall mean for the electrical stimulation group in the contralateral eye was not significantly different from the overall mean for the control group, ARC injected naloxone, subconjunctivally injected naloxone, and ARC injected saline all increased IOP in electrically stimulated animals. Subconjunctival saline had no effect. It is possible that the lack of statistical significance between the over-all means for control and electrically stimulated animals in the contralateral eye was because the difference between the 2 means, being less than that seen in the ipsilateral eye, was unable to achieve statistical significance.

When data from the 35–60 min period are averaged and the 2 eyes are compared (Table 2), IOP in the contralateral eye is 19.71 for control, 13.03 for electrical stimulation, 19.81 for ARC naloxone, 19.21 for ARC saline, 21.19 for subconjunctival naloxone, and 12.50 for subconjunctival saline. The average IOP after electrical stimulation for the ipsilateral eye for this time period is 8.57. By this method of looking at the data, unilateral electrical stimulation of the ARC is seen to decrease IOP in the contralateral eye, but to a lesser degree than the decrease seen in the ipsilateral eye. And both ARC and subconjunctival naloxone completely blocked the electrical stimulation-induced decrease in IOP (Table 2).

### The effects of μ agonist (DAMGO) injection into the ARC

**Figure 3:**
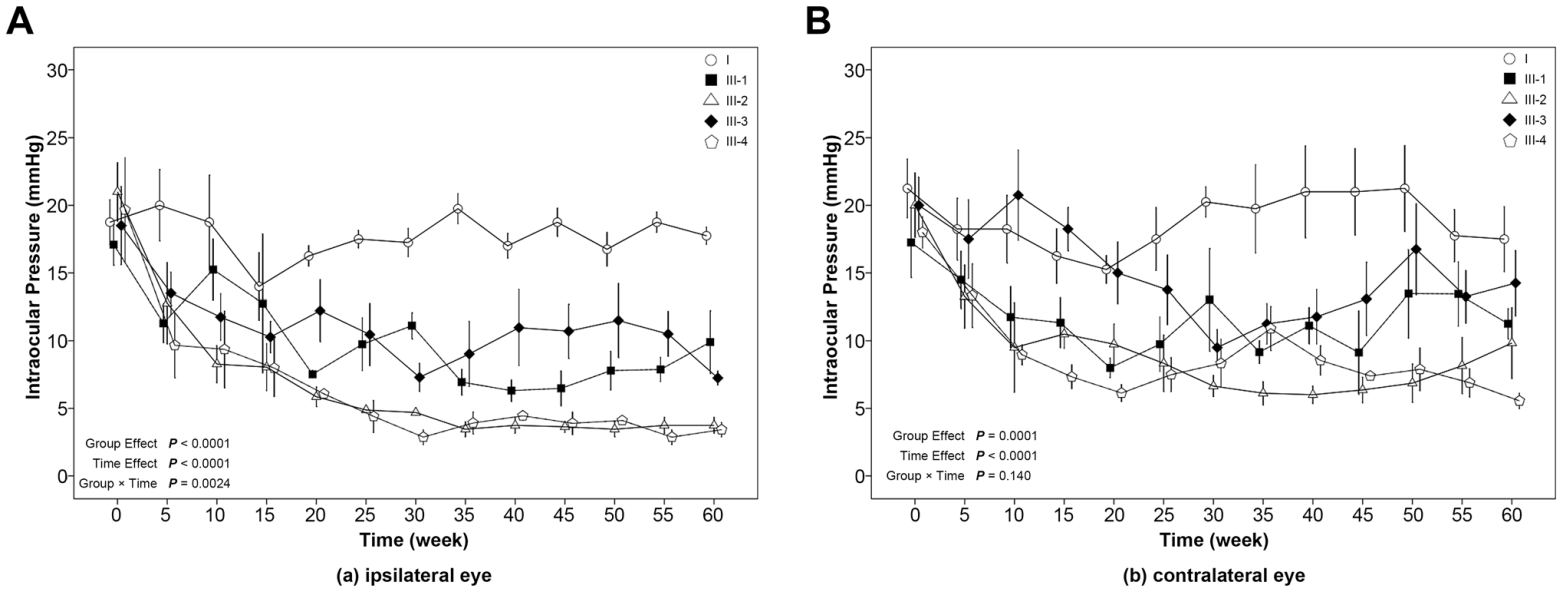
The effect of unilateral injection of the μ opioid receptor agonist, DAMGO, into the ARC on intraocular pressure in the ipsilateral eye (a) and the contralateral eye (b). The error-bar (mean ± SE) of each group is located at time points of 0, 5, 10, 15, 20, 25, 30, 35, 40, 45, 50, 55 and 60 minutes. A slight separation was made for error-bars at the same time points to avoid overlapping. I, control group; III-1, 2 µmol/µl DAMGO injection group; III-2, 4 µmol/µl DAMGO injection group; III-3, subconjunctival naloxone+2 µmol/µl DAMGO group; III-4, subconjunctival saline+2 µmol/µl DAMGO group.

For IOPs in the ipsilateral eye after μ agonist microinjection (Figure 3a), the group effect, time effect, and group×time were all significant (*P*<0.05). These results indicate that the changes in IOP over time were different among groups. The overall control group mean was significantly higher than the 2 µmol/µL (mean difference = 7.78 mmHg; *P*<0.001) and 4 µmol/µL DAMGO (mean difference = 11.07 mmHg; *P*<0.001), naloxone+2 µmol/µL DAMGO (mean difference = 6.71 mmHg; *P*<0.001), saline+2 µmol/µL DAMGO (mean difference = 11.43 mmHg; *P*<0.001) group means. If, as in the electrical stimulation data, we look at average IOP in the ipsilateral eye during the 35–60 min quasi-steady state period (Table 2), the IOPs are the following: control, 18.13; 2 µmol/µL DAMGO, 7.55; 4 µmol/µL DAMGO, 3.63; naloxone+2 µmol/µL DAMGO, 9.99; saline+2 µmol/µL DAMGO, 3.78. In other words, intra-ARC DAMGO elicits a dose-dependent decrease in IOP that is unaffected by subconjunctival naloxone and possibly decreased further by subconjunctival saline.

The IOPs for the contralateral eye (Figure 3b) showed both a group effect (*P* = 0.0001) and a time effect (*P*<0.0001). The overall control group mean was significantly higher than the 2 µmol/µL (mean difference = 7.08 mmHg; *P* = 0.006) and 4 µmol/µL DAMGO (mean difference = 9.53 mmHg; *P* = 0.0004) and saline+2 µmol/µL DAMGO (mean difference = 9.88 mmHg; *P* = 0.0005) group means. The naloxone+2 µmol/µL DAMGO group mean was significantly different from the saline+2 µmol/µL DAMGO group (mean difference = −5.68 mmHg; *P* = 0.037).

When the data was recalculated as average IOP for the 35–60 min period in the contralateral eye, the following IOPs were seen: Control, 19.71; 2 µmol/µL DAMGO, 11.27; 4 µmol/µL DAMGO 7.23; naloxone+2 µmol/µL DAMGO, 13.39; saline+2 µmol/µL DAMGO, 7.86. In other words, 2 and 4 µmol/µL decrease IOP in a concentration-dependent manner in both the ipsilateral and contralateral eye, but to a lesser extent in the contralateral eye. Subconjunctival naloxone has little effect on IOP and saline seems to magnify the IOP decrease in response to low dose DAMGO.

### The effects of δ agonist (DPDPE) injection into the ARC

**Figure 4:**
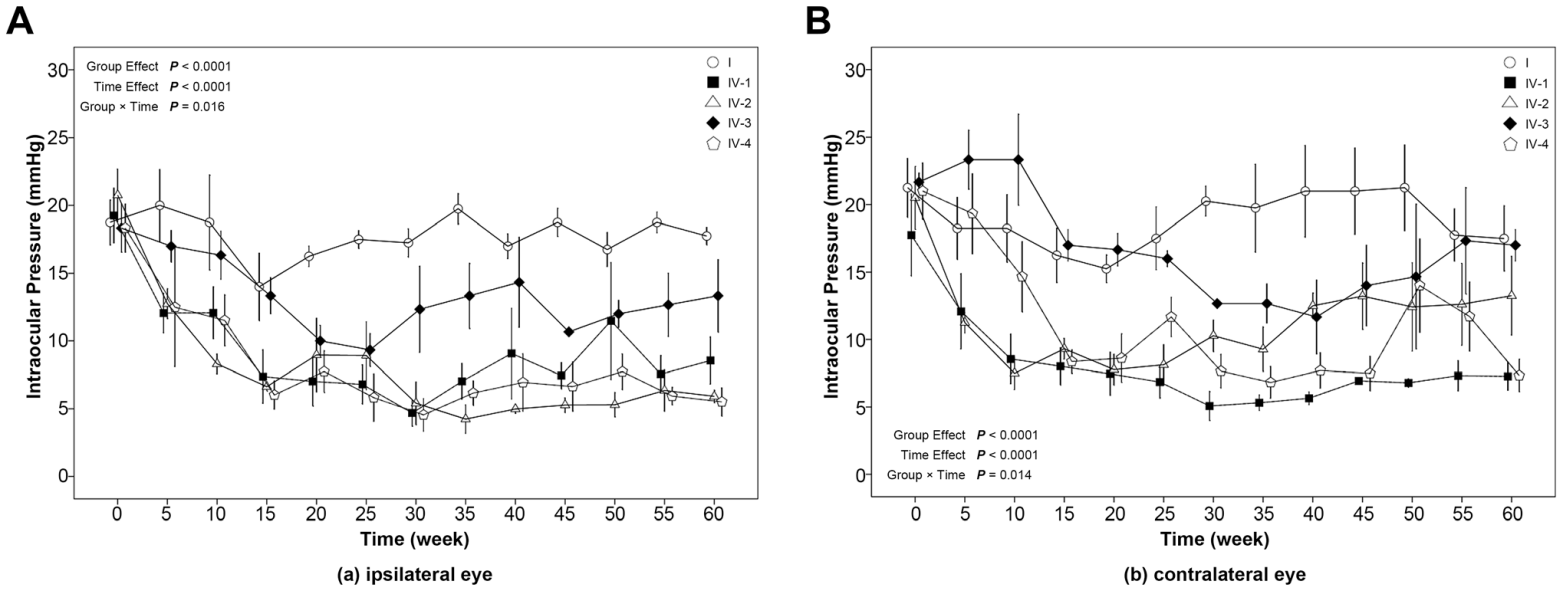
The effects of unilateral injection of the δ opioid receptor agonist, DPDPE. into the ARC on intraocular pressure for the ipsilateral eye (a) and the contralateral eye (b). The error-bar (mean ± SE) of each group was located at time points of 0, 5, 10, 15, 20, 25, 30, 35, 40, 45, 50, 55 and 60 minutes. A slight separation was made for error-bars at the same time point to avoid overlapping. I, control group; IV-1, 2 µmol/µg DPDPE group; IV-2, 4 µmol/µl DPDPE group; IV-3, subconjunctival naloxone+2 µmol/µg DPDPE group; IV-4, subconjunctival saline+2 µmol/µg DPDPE group.

For IOPs in the ipsilateral eye after δ agonist injection into the ARC (Figure 4a), the Group Effect, Time Effect, and Group×Time were all significant (*P*<0.05). This indicates that the change in IOP over time was different among groups. Regarding overall group means, all groups had IOPs that were significantly lower than control: 2 µmol/L DPDPE (mean difference = −8.53 mmHg; *P*<0.0001); 4 µmol/µL DPDPE (mean difference = −9.80 mmHg; *P*<0.0001); subconjunctival naloxone+2 µmol/µL DPDPE (mean difference = −4.48 mmHg; *P* = 0.015); subconjunctival saline+2 µmol/µL DPDPE (mean difference = −9.68 mmHg; *P*<0.0001). For the 35–60 min time period, average IOPs were as follows: Control, 18.13; 2 µmol/µL DPDPE, 8.52; 4 µmol/µL DPDPE, 5.34; subconjunctival naloxone+2 µmol/µL DPDPE, 12.72; subconjunctival saline+2 µmol/µL DPDPE, 6.49. In other words, for the δ agonist, in the ipsilateral eye a dose-response relationship is seen that is slightly, if at all, blocked by subconjunctival naloxone.

For IOPs in the contralateral eye (Figure 4b), the Group Effect, Time Effect, and Group×Time were all significant (*P*<0.05). Therefore, as in the ipsilateral eye, the change in IOP over time was different among groups. Regarding overall group means, compared to the control group, 2 µmol/µL DPDPE (mean difference = −10.78 mmHg; *P*<0.001), 4 µmol/µL DPDPE (mean difference = 7.48 mmHg; *P* = 0.002), and subconjunctival saline+2 µmol/µL DPDPE (mean difference = 7.607; P = 0.0034) groups showed significantly lower IOPs.

The 35–60 min averages for contralateral IOP were as follows: Control 19.71; 2 µmol/µL DPDPE, 6.55; 4 µmol/µL DPDPE, 12.21; subconjunctival naloxone+2 µmol/µL DPDPE, 14.56; subconjunctival saline+2 µmol/µL DPDPE, 9.17. Here 2 µmol of the δ opioid decreases IOP slightly more in the contralateral than in the ipsilateral eye, and subconjunctival naloxone partly blocks this response. But surprisingly, 4 µmol/µL DPDPE is less effective than 2 µmol/µL DPDPE in lowering IOP.

## Discussion

In the ipsilateral eye, electrical stimulation of the ARC caused a decrease in IOP that was blocked by naloxone injected into the ARC and by subconjunctival naloxone. ARC microinjection of either the selective μ or the selective δ opioid receptor agonist also decreased IOP in the ipsilateral eye, and did so in a dose-dependent manner. But subconjunctival naloxone had little or no effect in blocking the ARC μ or δ agonist-induced decrease in IOP.

In the contralateral eye, electrical stimulation of the ARC decreased IOP, but to a lesser extent than in the ipsilateral eye. Both ARC and subconjunctival naloxone completely blocked this milder decrease in IOP. Central administration of the μ agonist, DAMGO, produced a dose-dependent decrease in IOP in the ipsilateral eye, and also, but to a lesser extent, in the contralateral eye. Subconjunctival naloxone blocked this decrease very little, if at all in either the ipsilateral or contralateral eye.

Although the dose-response relationship to the δ agonist, DPDPE and relative lack of effect of naloxone in the ipsilateral eye were similar to what was seen with the μ agonist, the response in the contralateral eye differed, because the response to low dose DPDPE was greater in the contralateral eye than in the ipsilateral eye and was clearly blocked to some extent by subconjunctival naloxone. Also, the response to high dose DPDPE in the ipsilateral eye was smaller than the response to low dose DPDPE. In other words, the response to DPDPE showed dose-dependence in lowering IOP in the ipsilateral eye, but an “inverse dose-dependence” in lowering IOP in the contralateral eye.

Our data show the ARC to be involved in bilateral control of IOP in rabbits. Unilateral electrical stimulation of the ARC caused IOP reduction in both the ipsilateral and the contralateral eye. Microinjection of naloxone into the ARC to determine whether the effects of electrical stimulation of the ARC are related to activity of the opioid peptides in this nucleus showed naloxone to block the IOP decrease caused by electrical stimulation, a result suggesting that opioid peptide neurons in the ARC are involved in the reaction to electrical stimulation.

However, it is probable that the blockade after ARC naloxone injection of stimulation-induced IOP decrease was not due to naloxone itself, because ARC injection of saline also blocked the stimulation-induced IOP decrease. The reason for this anomalous result should be investigated further. It is possible that the use of artificial cerebrospinal fluid instead of saline for the microinjections, or changes in injection time or volume, would preserve the naloxone effect and eliminate the unexpected control effect.

We also examined whether peripheral release of opioid peptides was involved in the regulatory effect of the arcuate nucleus on IOP, and found that subconjunctival naloxone blocked the decrease in IOP caused by electrical stimulation. Therefore peripheral as well as ARC opioids are involved in the response to electrical stimulation of the ARC

Another indication that part of the regulation of IOP through the ARC is due to central opioid action was that unilateral microinjection of μ and δ opioid agonists into the ARC caused a dose dependent decrease in IOP for both agonists in the ipsilateral eye and, for the μ agonist, also in the contralateral eye. Subconjunctival injection of naloxone had little effect in inhibiting these responses to centrally injected opioid.

Results for unilateral stimulation or μ agonist injection in the ARC are fairly straightforward. Either unilateral stimulation or agonist injection elicit a bilateral response that is weaker in the contralateral eye. Subconjunctival naloxone partially blocks the response to ARC electrical stimulation, but not to ARC injection of μ agonist.

Although the effect of ARC injection of δ agonist on IOP in the ipsilateral eye is similar to the effect of the μ agonist, the effect on the contralateral eye is quite different. Here the low dose of agonist produces a bigger response than the high dose. One possible explanation would be that at the high dose, the δ agonist activated another receptor in the ARC that antagonized the low dose action. But if this were the explanation, this central effect should be seen in both eyes. Another result of ARC δ agonist injection that needs explanation is that although subconjunctival naloxone had little or no effect on the δ agonist-induced IOP decrease in the ipsilateral eye, it produced a clear partial inhibition of the response to low dose δ agonist in the contralateral eye. This result implies that for the δ agonist, local opioid release was probably not involved in the ipsilateral response but was, somehow, involved in the contralateral response. Delta agonists are known to have a number of different cellular effects, including effects on glutamate receptors [11]. Study of these effects may provide an explanation for our results.

Opioids have long been known to be involved in the control of IOP. Morphine, either intravenous or intraocular injections, decreases IOP in rabbits [2]. Reduction of the aqueous flow rate through activation of μ-3 opioid receptors and increased production of NO has been reported to be involved morphine's action [10]. The μ agonist DAMGO and the δ agonist DPDPE have been shown to deplete melatonin levels in the ciliary body, an action that causes IOP to fall [12]. μ and δ agonists have also been shown to decrease the excitatory post-synaptic current in rat brain slices [11].

Kappa agonists are also involved in regulation of IOP. Topical application of a selective κ opioid agonist, bremazocine, has been shown to reduce aqueous humor flow rate presumably by increasing atrial natriuretic peptide levels [13], [14], [15]. A second selective κ agonist, spiradoline, was found to increase NO levels in both ciliary and trabecular meshwork cells, an action that would decrease aqueous humor production and increase its outflow [3]. Acupuncture electrostimulation was found to decrease aqueous humor flow rate through activation of the sympathetic nervous system. But in the acupuncture experiment, IOP remained low after the aqueous humor flow rate and aqueous humor norepinephrine and dopamine levels had returned to normal, and the time course of the decreased IOP was related to increased aqueous humor endorphin levels [7].

However, there are many different types of neurons and neurotransmitters in the ARC, and there could be a number of currently unknown downstream effects of ARC stimulation and opioid microinjection. Glutamate, in particular, has been implicated in the downstream action of μ and δ opioids [11], [16] and in retinocollicular neurotransmission in the eye [17].

In trying to relate the current results to the work of others, however, one must take into account that the studies of others covered the time period from 0.5–1 h to 4–6 h after the treatment was applied. Our study, in contrast, covered the time period from 5 min to 1 h after treatment. Many of the changes reported by others were not visible until later times than the time period covered in this study. One observation seen in our study in the 0–30 min time period, the time period not covered by others, was that subconjunctival naloxone did not inhibit the effects of electrical stimulation until this time period had passed. Perhaps the presence of an endogenous opioid system connected to intracellular signaling pathways made the antagonist take a longer time to produce blockade.

Opioid neurons and receptors are present locally in the eye. Selbach [18] used antibodies to enkephalin and nociceptin to stain the anterior segment sections of rat eye and found that a small number of nerve fibers in the base between the iris root and the ciliary process were stained. The epithelium of the ciliary body, ciliary muscle, and papillary muscles were not stained at all [18]. However, Russell-Randall [3] reported κ opioid receptors to be present in rabbit non-pigmented ciliary epithelial cells, and in human ciliary body and trabecular meshwork cell lines.

To date, there has been no in-depth study on the relationship between the arcuate nucleus and IOP, The current study is a qualitative study to investigate whether or not the hypothalamic arcuate nucleus was involved in control of IOP in rabbits. Our results showed that the hypothalamic arcuate nucleus participates in the central negative regulation of the IOP in rabbits. However, the nerve fiber connections in the brain are complex and we were unable to determine whether the reaction in the central nervous system is through the endogenous opioid system directly or through an indirect connection between the arcuate nucleus and other neurons. In the future, magnetic resonance or radionuclide scanning could be used to identify a series of eye-related responses caused by excitation of the arcuate nucleus.

One limitation of the study is that we did not include a sham group in which the surgery was performed. However, in preliminary experiments we did compare animals between control and sham groups and had shown no differences. Notably, the rabbits in the groups II, III, and IV underwent the same operation. Any effects due to surgery should have been the same for II, III, and IV groups. A second limitation is that we did not examine possible dose effects of naloxone or use ARC microinjection in the μ and δ agonist protocols. The partial blockade or lack of blockade seen in some of our results could be due to the fact that the antagonist was not administered long enough or that the concentration used was not high enough. The use of central microinjection of antagonist in the μ and δ agonist protocols might have changed the results if afferent opioid input to the ARC or an independent regulatory pathway were involved [19], [20]. For example, Ludwig [20] reported that GABAergic projection from the arcuate nucleus to the supraoptic nucleus in the rat could be divided as part of the inhibitory pathway arising from or passing through the arcuate nucleus to the supraoptic nucleus and mediated by the neurotransmitter gamma-aminobutyric acid (GABA). However, the post-inhibitory excitation induced by arcuate stimulation is not a rebound response, but appears to involve an independent excitatory pathway. Other limitations of the current study are that limitations in the procurement of animals prevented us from using an optimal protocol design, that we did not use selective antagonists for the δ and μ agonists, and that a longer time period for IOP measurement was not included. Although we have HE stain anatomic slice evidence to show the injections to ARC is correct, we still need more solid evidence to prove the accurate localizations of ARC such as gonadotropin-releasing hormone (GnRH) -specific immunohistochemical staining for ARC.

Does release of opioid peptides or other neurotransmitters occur locally in the eye? The observation that locally administered opioid antagonist had different effects on ARC electrical stimulation and opioid microinjection suggest the involvement of other neurotransmitters, for example, melatonin, ANP, NO, and possibly glutamate. However, at present we are still unable to determine whether all of these neurotransmitters play a role, which neurotransmitters play a major role, or the relationship between them. Based the response to unilateral stimulation of the ARC is stronger in the ipsilateral eye than the contralateral eye, we speculate that nerve fibers projecting from the arcuate nucleus or at least from the hypothalamus to the eye are present and that when the arcuate nucleus is excited (electrical stimulation or selective agonist), a small number of nerve fibers in the eye release a small amount of opioid peptides or perhaps other neurotransmitters, a release that regulates the aqueous humor flow through ANP, NO or some other unknown substance, reducing the IOP. At the same time, the contralateral arcuate nucleus in is excited, causing an IOP decrease in the contralateral eye.

It is worth noting that pharmacological evidence based on the effect of agonists and antagonists together with physiological findings (electric stimulation of brain areas) may not be sufficient to demonstrate the involvement of a given receptor, because the probability of off-target effects has to be taken into account [21]. Manipulating the expression levels of receptors using molecular techniques and genetic alterations represents the most specific pharmacologic strategy available today. This approach has been successfully applied, for example, to demonstrate the involvement of dopamine D3 receptor subtype in IOP regulation [22] and might help, in the near future, in elucidating the role of the opioid system.

Central regulation of IOP may be similar to central regulation of blood pressure [18]: In blood pressure regulation, the rostral ventrolateral medulla contains the nuclei that maintain stable arterial blood pressure and control vascular tone [23]. Nerve centers acting on the heart or blood vessels are also distributed at all levels from the cerebral cortex to the spinal cord. The ‘cardiovascular center’ is a coherent whole. However, the final upstream control of sympathetic nervous system output is through the pressor area of the ventral surface of the medulla oblongata. It cannot be determined whether the arcuate nucleus is the “final upstream regulator” of IOP based on our study results. We can only say that it is important in regulation the downstream events that determine IOP, and we are not sure whether opioid peptides play a role in IOP regulation similar to the role of norepinephrine in blood pressure regulation. However, we hope that we can continue to explore this possibility. Perhaps there is some unspecified type of receptor in the eye or elsewhere that acts in a similar manner to the aortic sinus and the carotid body and communicates continuously a central nerve center (arcuate nucleus or other) to regulate the IOP through a feedback loop, allowing the IOP to fluctuate within a normal range. After surgery, the IOP in the surgically treated eye is decreased and the total requirement for the negative regulation by opioid peptides is decreased. The control center then automatically reduces the secretion of opioid peptide, thereby causing IOP fluctuations in the contralateral eye.
